# *Balitora tiandengensis* (Teleostei, Balitoridae), a new species of cave-dwelling loach from Guangxi, China

**DOI:** 10.3897/zookeys.1267.166354

**Published:** 2026-01-30

**Authors:** You Nong, Yuan Fang, Jin-Yue Li, Qiu-Jun Wei, Chuan-Gui Xu, Gui-Yuan Wei

**Affiliations:** 1 Guangxi Key Laboratory of Traditional Chinese Medicine Quality Standards, Guangxi Institute of Chinese Medicine & Pharmaceutical Science, No. 20–1 Dongge Road, Nanning, Guangxi, China Guangxi Normal University Guilin China; 2 Guangxi Normal University, No.1 Yanzhong Road, Guilin, 541006, Guangxi, China Guangxi Key Laboratory of Traditional Chinese Medicine Quality Standards, Guangxi Institute of Chinese Medicine & Pharmaceutical Science Nanning China

**Keywords:** Karst cave, loach, morphology, phylogeny, taxonomy

## Abstract

*Balitora tiandengensis* (Teleostei, Balitoridae), a new species collected from a karst cave at Dukang Town, Tiandeng County, Guangxi, China is described and illustrated on the basis of morphological and molecular evidence. Phylogenetic trees reconstructed based on 70 sequences of two mitochondrial (COI and Cyt b) and three nuclear genes (RAG1, IRBP, and EGR2B) show that the new species represents an independent evolutionary lineage. Morphologically, *Balitora tiandengensis* Nong & Wei, **sp. nov**. can be distinguished from the other species currently assigned to the genus *Balitora* by its dorsal fin iii-9, anal fin i-6, pectoral fin v-12, and caudal-fin 19 branched rays. The lips exhibit no complex folding or thickening, maintaining a relatively thin and simple structure. Upper and lower lips connected at corner of mouth, upper lip with a row of inconspicuous papillae, and lower lip thin. Dorsal fin long, 16.7–19.4% of standard length (SL), anal fin short, 15.0–16.7% of SL, distal margin truncated, origin close to the anus and far from the caudal-fin base, pectoral fin 18.4–20.5% of SL, pelvic fin moderately developed, distal margin rounded, 15.1–16.7% of SL. Anus 72.5%–73.3% distance from posterior end of the pelvic fin base to the anal fin origin. The new species will help to define the new distribution of the genus *Balitora* in Guangxi, China.

## Introduction

The genus *Balitora*, Gray, 1830 was established with *Balitora brucei* Gray, 1930 as the type species, originally placed in Cobitidae but it is currently the type genus for the family Balitoridae (Kottelat and Chu 1988; Fricke et al. 2025). The genus *Balitora* has long been the subject of taxonomic controversy, with different taxonomic schemes proposed based on morphological differences (Zheng et al. 1982; Zheng and Zhang 1983; Li and Chen 1985). At first, it was recognized by one pair of maxillary barbels, a character that distinguishes *Balitora* from other genera (Chen 1978). Kottelat (1988) and Kottelat and Chu (1988) reviewed this genus, revealed the unreliability of maxillary-barbel number in defining the three Balitorid genera, *Balitora*, *Hemimyzon*, and *Sinohomaloptera*, and proposed that *Sinohomaloptera* be a junior subjective synonym of *Balitora*. Based on three or more unbranched pelvic fin rays, *B. elongata* Chen & Li, 1985, *B. nujiangensis* Zhang & Zheng, 1983, *B. pengi* Huang, 1982, and *B. tchangi* Zheng, 1982 were placed in *Hemimyzon* Regan 1911, while species with two branched pelvic fin rays were placed in *Balitora*, namely *B. kwangsiensis* (Fang, 1930), *B. lancangjiangensis* (Zheng, 1980), and *B. longibarbata* (Chen, 1982) (Kottelat and Chu 1988). However, Chinese scholars did not embrace the proposal by Kottelat and Chu (1988), and continued to classify species within the genus *Balitora* based on the count of maxillary barbels as having one or two pairs of maxillary barbels at each corner of mouth. So far, no in-depth phylogenetic studies of Balitoridae species have been conducted.

Nevertheless, previous morphology-based studies have not resolved the phylogenetic relationships between *Balitora*, *Hemimyzon*, and *Sinohomaloptera*, with various studies concluding that the phylogenetic position of the genus *Balitora* is unclear and may not be monophyletic (Šlechtová et al. 2007; Liu et al. 2012a; Kumkar et al. 2016; Tao et al. 2019; Shao et al. 2020). The genus *Balitora* includes both surface-dwelling and cave-adapted species (Liu et al. 2012b; Luo et al. 2023). To date, the classification of the genus remains controversial, although Luo et al. (2023) showed clearly in their phylogenetic analysis that *Balitora* is not a monophyletic and contains three distinct clades with significant genetic divergence among species, but also suggested that more evidence was needed to further clarify the taxonomic composition of the genus *Balitora*.

In this study, we followed the latest taxonomic scheme for the genus *Balitora* (Balitoridae, Cypriniformes) as proposed by Luo et al. (2023), which integrated molecular phylogenetic analyses based on two mitochondrial (COI and Cyt b) and three nuclear genes (RAG1, IRBP, and EGR2B) with geometric morphometrics to resolve longstanding taxonomic controversies. According to the Catalog of Fishes (Fricke et al. 2025), the genus currently comprises 22 recognized species, of which twelve species are distributed in China, namely *B. anlongensis* Luo, Chen, Zhao, Yu, Lan & Zhou, 2023, *B. brucei*, *B. dehouensis* Lei, Pu & Yang, 2025, *B. elongata*, *B. kwangsiensis*, *B. lancangjiangensis*, *B. longibarbata*, *B. ludongensis* Liu & Chen, 2012, *B. nantingensis* Chen, Cui & Yang, 2005, *B. scyphus* Endruweit, 2025, *B. tchangi*, and *Balitora yingjiangensis* (Chen, 2006), which was previously mistakenly classified as *Hemimyzon*, and was re-described through examination of type specimens and collection of topotypic specimens from the Jieyanghe River, a tributary of the Irrawaddy River in Nabang Town, Yingjiang County, Yunnan Province, China (Liu et al. 2012b; Luo et al. 2023; Endruweit 2025; Lei et al. 2025a, 2025b).

During our field surveys for plant resources in Tiandeng County, Guangxi in December 2024, we unexpectedly collected five specimens of the genus *Balitora* in a karst cave. After consulting relevant literature (Kottelat and Chu 1988; Kottelat 1988; Chen et al. 2005; Nguyen 2005; Conway and Mayden 2010; Liu et al. 2012b; Luo et al. 2023; Lei et al. 2025a, 2025b) and comparing with relevant specimens, we confirm that this unusual fish represents a new species, which is described below. In addition, all five specimens were collected from the same karst cave water system, and this species has not been found in open surface waters outside the cave. Their eye diameter is small and light-sensitive, so we define it as a new species of cave-dwelling loach according to the study of Culver and Pipan (2019).

## Material and methods

### Sampling and morphological measurement

Five specimens representing a new species, were collected from Dukang Town, Tiandeng County, Guangxi Zhuang Autonomous Region, China for morphological comparison and two of them were sampled for genetic analysis (vouchers WGY2024121901 and WGY2024121904). All specimens were then fixed in 10% buffered formalin and later transferred to 70% ethanol for preservation. Muscle samples used for molecular analysis were preserved in 95% alcohol and stored at −20 °C. All specimens are deposited at Guangxi Institute of Chinese Medicine & Pharmaceutical Science (**GXMI**), Nanning City, Guangxi Zhuang Autonomous Region, China under the holotype number WGY2024121901 and paratypes numbers, WGY2024121902–WGY2024121905.

The new species was described, based on field observations that were made in December 2024 and examination of specimens at GXMI. Other related *Balitora* species examined during this study are based on data from the National Specimen Information Infrastructure (http://nsii.org.cn/2017/), FishBase (https://fishbase.mnhn.fr/), and National Animal Specimen Resource Center (http://museum.ioz.ac.cn/index.html). The morphological data of 19 species of the genus *Balitora* comes from relevant literature (Liu et al. 2012b; Luo et al. 2023; Lei et al. 2025a, 2025b). All measurements of the new species were taken on the left side of the fish specimens, point to point with digital calipers at 0.1 mm accuracy. Counts and proportional measurements follow Tang et al. (2012).

### Molecular phylogenetic analysis

The genomic DNA was extracted from muscle tissues by standard phenol chloroform methods (Sambrook and Russell 2001). Then two tissue samples used for molecular analysis were amplified and sequenced for two mitochondrial genes and three protein-coding genes: mitochondrial gene cytochrome b (Cyt b), cytochrome oxidase subunit 1 (COI), recombination activating gene 1 (RAG1), retinoid binding protein (IRBP), and early growth response protein 2B (EGR2B) as in Luo et al. (2023). Primer sequences are shown in Suppl. material 1. Both the amplification and sequencing were completed in Beijing Ruijie Gene Technology Co., Ltd (Beijing, China). All sequences were aligned using MAFFT v. 7.490 (Katoh and Standley 2013). The sequences after alignment were connected in series using the “Concatenate” tool in Genious Prime v. 2021.1.1 (Kearse et al. 2012). The aligned matrices were automatically trimmed using trimAL v. 1.4 (Capella-Gutiérrez et al. 2009) to remove non-conservative sequence parts. Maximum likelihood phylogenies were inferred using IQ-TREE v. 2.2.0 (Nguyen et al. 2014) under the GTR+I+G4+F model for 1000 ultrafast bootstraps (Minh et al. 2013). Vouchers of specimens and GenBank accession numbers for this study are presented in Table 1.

**Table 1.** Localities, voucher information, and GenBank numbers for all samples used in this study.

ID	Species	Locality (* type localities)	Voucher	Cyt b	COI	RAG1	IRBP	EGR2B
1	*Balitora anlongensis*	Xinglong Town, Anlong County, Guzihou, China	GZNU20230215018	OQ754144	OQ784688	OQ754158	OQ754154	OQ754149
2	*Balitora anlongensis*	Xinglong Town, Anlong County, Guzihou, China	GZNU20230215019	OQ754145	OQ784690	OQ754159	OQ754155	OQ754150
3	*Balitora anlongensis*	Xinglong Town, Anlong County, Guzihou, China	GZNU20230215020	OQ754143	OQ784689	OQ754157	OQ754152	OQ754148
4	*Balitora annamitica*	–	–	–	–	EF056359	–	–
5	*Balitora brucei*	–	CIFEFGB-Bb-02	MK732323	MK388804	–	–	–
6	*Balitora brucei*	–	HSLBB	–	KJ774109	–	–	–
7	*Balitora chipkali*	India, Karnataka, Ramnagar	BNHS FWF 193	KU378016	KU378003	–	–	–
8	*Balitora chipkali*	India, Karnataka, Kamra, Joida	WILD-15-PIS-230	KU378017	KU378004	–	–	–
9	*Balitora elongata*	Menglun, Yunnan Province, China	IHB0301053	DQ105218	–	–	–	–
10	*Balitora elongata*	Xishuangbanna, Yunnan Province, China	IHB0301030	DQ105217	–	–	–	–
11	*Balitora elongata*	–	cyp74	–	–	KP695617	KP695065	–
12	*Balitora jalpalli*	–	KUFOS-19-AN-BA-34.1	–	MT216524	–	–	–
13	*Balitora kwangsiensis*	Yuanjiang, Yunnan Province, China	IHB0805545	JN177004	–	JN177060	–	–
14	*Balitora kwangsiensis*	–	GZNU20230215022	OQ754147	–	OQ754161	OQ754153	–
15	*Balitora kwangsiensis*	Yuanjiang, Yunnan Province, China	IHB0805546	JN177006	JN177071	JN177058	–	–
16	*Balitora kwangsiensis*	Yuanjiang, Yunnan Province, China	IHB0805547	JN177005	JN177072	JN177059	JN177276	JN177248
17	*Balitora kwangsiensis*	/	/	DQ105216	–	–	–	–
18	*Balitora laticauda*	India, Maharashtra, Venegaon	WILD-12-PIS-019	KU378007	KU377994	–	–	–
19	*Balitora laticauda*	India, Maharashtra, Venegaon	ZSI-WRC P/2849	KU378008	KU377995	–	–	–
20	*Balitora ludongensis*	Jingxi City, Guangxi Province, China	GZNU20230215023	OQ754141	–	–	–	–
21	*Balitora ludongensis*	Jingxi City, Guangxi Province, China	GZNU20230215024	OQ754142	–	–	–	–
22	*Balitora ludongensis*	Jingxi City, Guangxi Province, China	SCAU-20190805001	MT157616	MT157616	–	–	–
23	*Balitora meridionalis*	–	–	–	–	KP322550	–	–
24	*Balitora mysorensis*	India, Karnataka, Hattihole	WILD-15-PIS-231	KU378018	KU378005	–	–	–
25	*Balitora mysorensis*	India, Karnataka, Hattihole	BNHS FWF 197	KU378019	KU378006	–	–	–
26	*Balitora tiandengensis* sp. nov.	Dukang Town, Tiandeng County, Guangxi, China*	WGY2024121901	PX672557	PX651850	PX677672	PX677670	PX678864
27	*Balitora tiandengensis* sp. nov.	Dukang Town, Tiandeng County, Guangxi, China*	WGY2024121904	PX672558	PX651849	PX677673	PX677671	PX678865
28	*Balitoropsis ophiolepis*	–	Vial 2006-0588	–	KR052868	KP322540	–	–
29	*Balitoropsis zollingeri*	–	Vial 2005-0948	–	KR052865	KP322535	–	–
30	*Beaufortia liui*	Panzhihua, Sichuan Province, China	IHB1004172	JN177009	JN177069	–	–	–
31	*Beaufortia szechuanensis*	Zhaotong, Yunna Province, China	IHB0709025	JN177007	JN177067	JN177061	JN177281	JN177253
32	*Bhavania australis*	–	KUFOS.2019.12.54	MT002547	MT002512	MT002571	–	–
33	*Formosania chenyiyui*	Changting, Fujia Province, China	IHB0301051	MK135435	MK135435	–	–	–
34	*Ghatsa montana*	–	KUFOS-19-RR-GA-39.1	–	MT216529	–	–	–
35	*Ghatsa pillaii*	–	KUFOS-19-AN-GA-40.1	–	MT216530	–	–	–
36	*Ghatsa santhamparaiensis*	–	KUFOS-19-AN-GA-42.1	–	MT216532	–	–	–
37	*Hemimyzon formosanus*	–	cyp903	AY392484	KU943001	–	KP695096	–
38	*Hemimyzon nujiangensis*	Nujiang, Yunnan Province, China	/	–	KM610757	–	–	–
39	*Hemimyzon nujiangensis*	Nujiang, Yunnan Province, China	ihb201305588	–	KM610756	–	–	–
40	*Hemimyzon taitungensis*	Taiwan Province, China	–	KX056121	–	–	–	–
41	*Hemimyzon yaotanensis*	Mudong, Chongqing City, China	IHB0809019	JN176994	–	JN177053	–	–
42	*Homaloptera bilineata*	–	–	–	–	KP322549	–	–
43	*Homaloptera confuzona*	–	CBM: ZF 11705	NC_033955	NC_033955	KP322543	–	–
44	*Homaloptera johorensis*	–	CBM: ZF 12286	NC_033952	NC_033952	–	–	–
45	*Homaloptera leonardi*	–	–	AB242165	AB242165	EU711130	FJ197076	AB531164
46	*Homaloptera montana*	–	NBFGR:8118E	–	HQ219123	–	–	–
47	*Homaloptera ocellata*	–	CBM:ZF 12287	NC_033953	NC_033953	KP322539	–	–
48	*Homaloptera ogilviei*	–	–	NC_031635	NC_031635	–	–	–
49	*Homaloptera parclitella*	–	–	NC_031634	NC_031634	EU409610	EU409668	EU409732
50	*Homaloptera sexmaculata*	–	–	–	ON903153	KP322545	–	–
51	*Homalopteroides smithi*	–	CBM: ZF 12281	NC_033957	NC_033957	KP322546	–	–
52	*Homalopterula gymnogaster*	–	Vial SN25	–	–	KP322554	–	–
53	*Jinshaia abbreviata*	Guizhou Province, China	IHB0709424	JN176992	JN177228	N177180	JN177274	JN177249
54	*Jinshaia sinensis*	Chongqing City, China	IHB0301068	JN176985	JN177117	JN177044	–	–
55	*Lepturichthys dolichopterus*	Jianou, Fujian Province, China	IHB0706070	GU084245	JN177091	JN177029	–	–
56	*Lepturichthys fimbriata*	Jinkou, Hubei Province, China	IHB0803128	GU084229	JN177102	JN177182	JN177272	JN177251
57	*Lepturichthys fimbriata*	Mudong, Chongqing City, China	IHB0301070	JN176942	JN177139	JN177020	JN177273	–
58	*Metahomaloptera omeiensis*	Mudong, Chongqing City, China	IHB0301071	JN177000	JN177080	JN177041	–	–
59	*Myxocyprinus asiaticus*	Mudong, Chongqing City, China	IHB0809033	JN176936	–	JN177063	–	JN177263
60	*Sinogastromyzon hsiashiensis*	Taoyuan, Hunan Province, China	IHB1004171/IHB030105	JN176997	JN177109	JN177054	–	–
61	*Sinogastromyzon nantaiensis*	Taiwan Province, China	ASIZP0806662	–	KU943003	–	–	–
62	*Sinogastromyzon puliensis*	–	–	FJ605359	FJ605359	–	–	–
63	*Sinogastromyzon sichangensis*	Cishui, Guizhou Province, China	IHB0400184	JN176998	JN177078	JN177040	KP695068	KP694496
64	*Sinogastromyzon sichangensis*	–	P4	OQ754146	–	OQ754160	OQ754156	OQ754151
65	*Sinogastromyzon szechuanensis*	Neijiang, Sichuan Province, China	20170920BB03	MN241814	MN241814	–	–	–
66	*Sinogastromyzon tonkinensis*	Yuanjiang, Yunnan Province, China	IHB0805543	JN177002	JN177074	JN177056	JN177277	JN177247
67	*Sinogastromyzon wui*	Zhaoping, Guangxi Province, China	IHB0400321	JN177001	JN177076	–	–	–
68	*Travancoria elongata*	–	–	–	MT216535	–	–	–
69	*Travancoria jonesi*	–	KUFOS-19-AN-TR-49.1	–	MT216539	–	–	–
70	*Vanmanenia caldwelli*	Wuyishan, Fujian Province, China	IHB0706028	JN177011	JN177232	JN177178	JN177280	JN177252

## Results

### Molecular phylogenetic analysis

**Figure 1.**
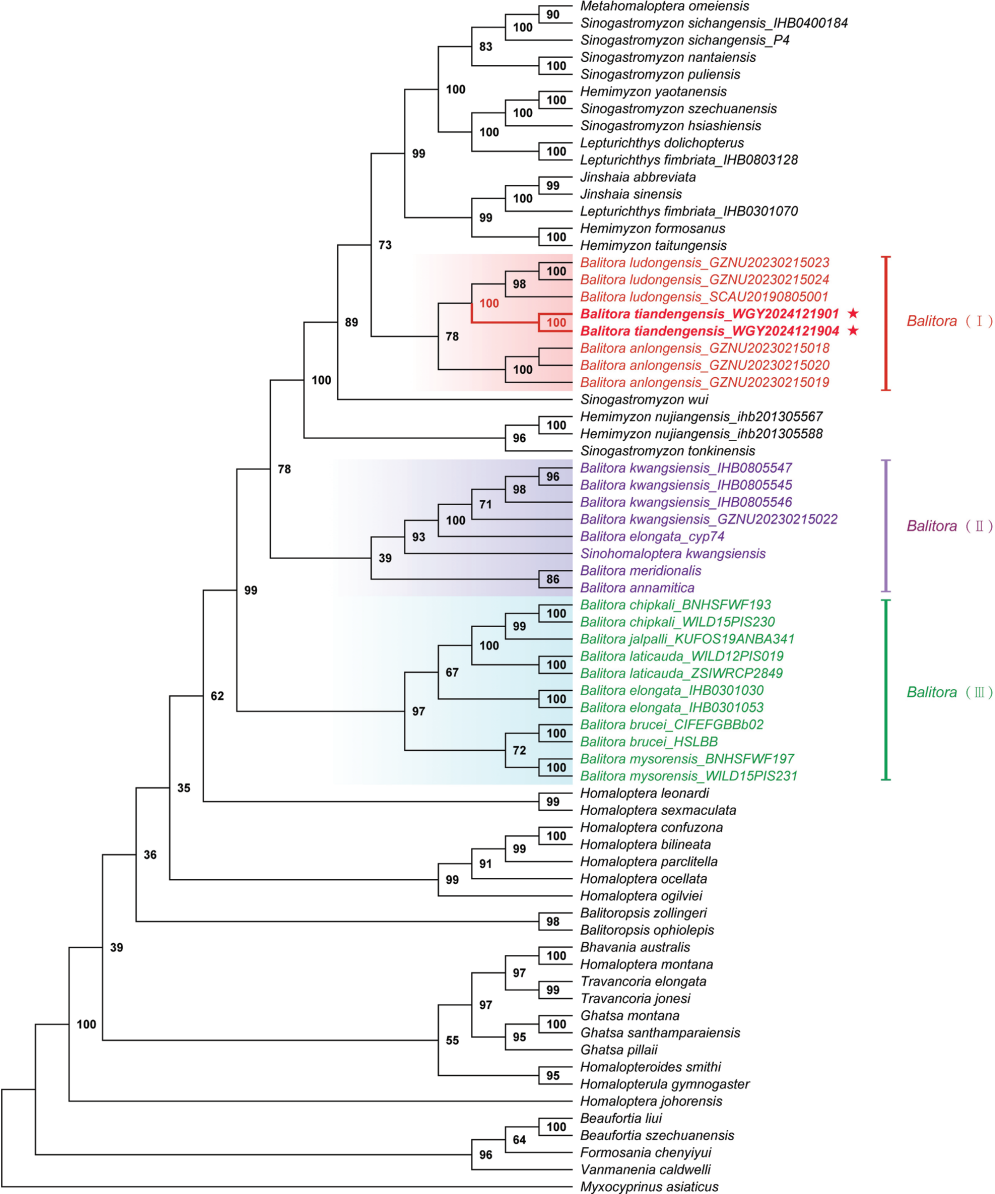
Maximum likelihood phylogenetic reconstruction of *Balitora tiandengensis* sp. nov. and its related species based on combined dataset COI+Cyt b+RAG1+IRBP+EGR2B. The bootstrap supports from maximum likelihood (ML) analyses are indicated alongside the corresponding nodes.

The ML tree based on two mitochondrial genes and three nuclear genes (Fig. 1) shows that *Balitora* is not a monophyletic and contains three distant clades. Clade I, including, *B. anlongensis*, *B. ludongensis*, and *B. tiandengensis*; Clade II, including, *B. annamitica* Kottelat, 1988, *B. elongata* Chen & Li, 1985, *B. kwangsiensis*, and *B. meridionalis* Kottelat, 1988; and Clade III, including *B. brucei* Gray, 1830, *B. chipkali* Kumar, Katwate, Raghavan & Dahanukar, 2016, *B. elongata* Chen & Li, 1985, *B. jalpalli* Raghavan, Tharian, Ali, Jadhav & Dahanukar, 2013, *B. laticauda* Bhoite, Jadhav & Dahanukar, 2012, and *B. mysorensis* Hora, 1941. *Balitora tiandengensis* sp. nov. formed sister groups with *B. ludongensis*. The smallest and biggest uncorrected p-distance (%) between *Balitora tiandengensis* sp. nov. and other species of the genus *Balitora* were 0.6% with *B. jalpalli* and 2.51% with *B. anlongensis* (Table 4). It can be distinguished by the combination of the following morphological characters: dorsal fin rays iii, 9, anal fin rays i, 6, pectoral fin rays v, 12, pelvic fin rays ii, 7, lateral line scales 69–75. The combination of distinct morphological characteristics and phylogenetic evidence supports the recognition of this previously unidentified population as a novel taxonomic entity. Thus, the population at this locality represents an independently evolved lineage and is described as a new species, *Balitora tiandengensis* sp. nov.

### Taxonomy account

#### Balitora tiandengensis

Taxon classificationAnimaliaCypriniformesBalitoridae

Nong & Wei sp. nov.

0651CEAB-4DE3-5001-9EA6-B5618F8BBF7A

https://zoobank.org/A088AD1B-5C8B-476F-8E8C-D38D5BDB6AEE

**Figure 2.**
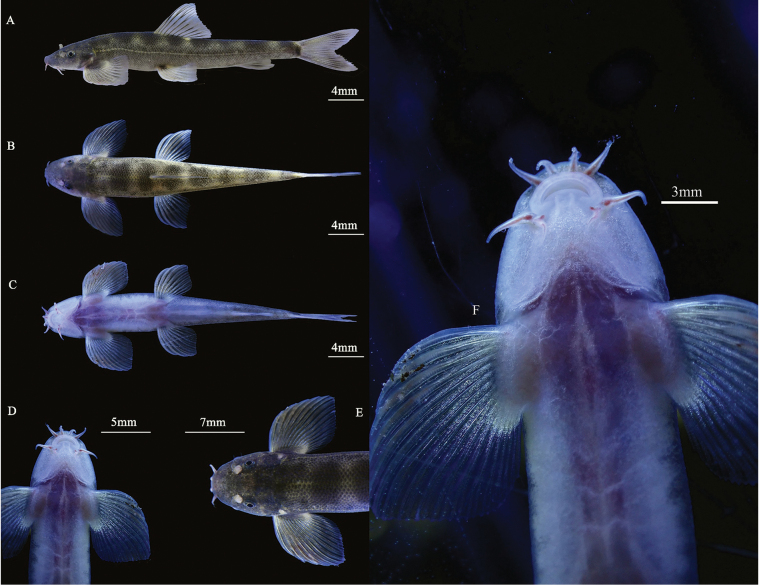
Morphological characters of holotype (WGY2024121901) of *Balitora tiandengensis* sp. nov. **A**. Lateral view; **B**. Dorsal view; **C**. Ventral view; **D**. Ventral side view of head; **E**. Dorsal side view of head; **F**. Close up photo of the mouth.

**Figure 3.**
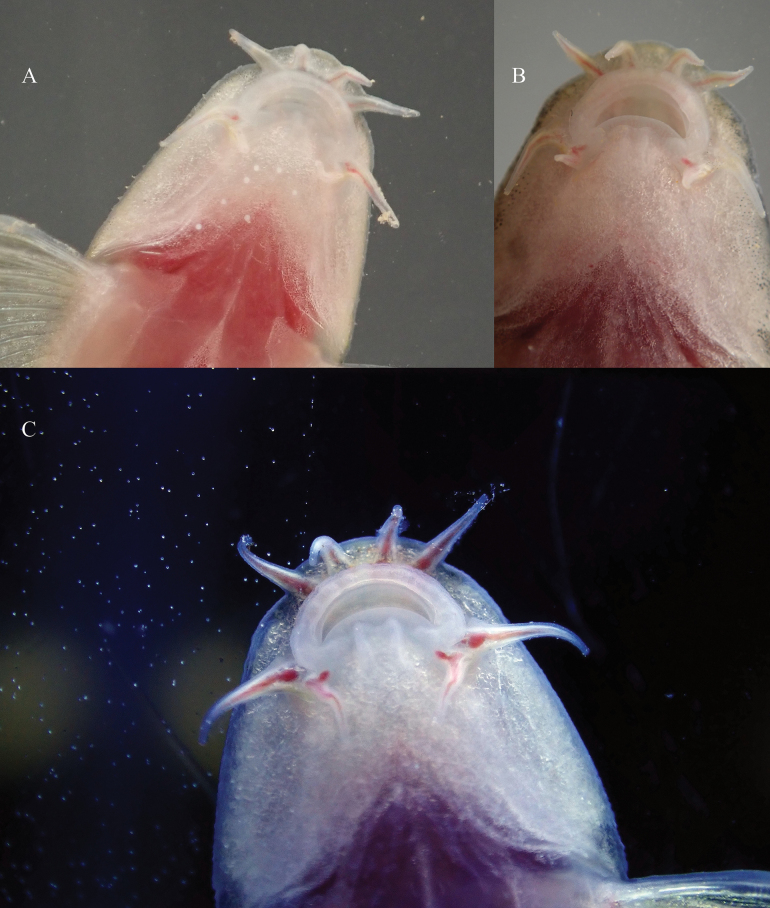
Oral morphology of *Balitora tiandengensis* sp. nov. **A**. Holotype (WGY2024121901); **B**. Paratype (WGY2024121902); **C**. Paratype (WGY2024121904).

**Figure 4.**
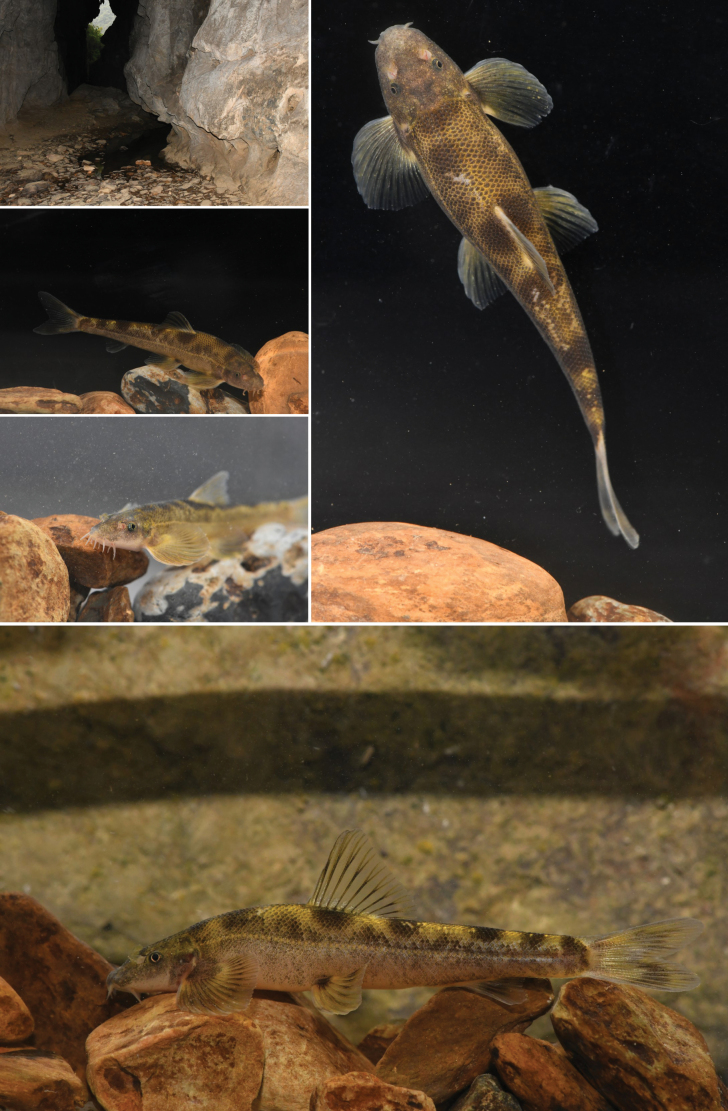
Habitat and photographs of *Balitora tiandengensis* sp. nov. in life.

Figs 2, 3, 4, Table 2

##### Type material.

**Figure 5.**
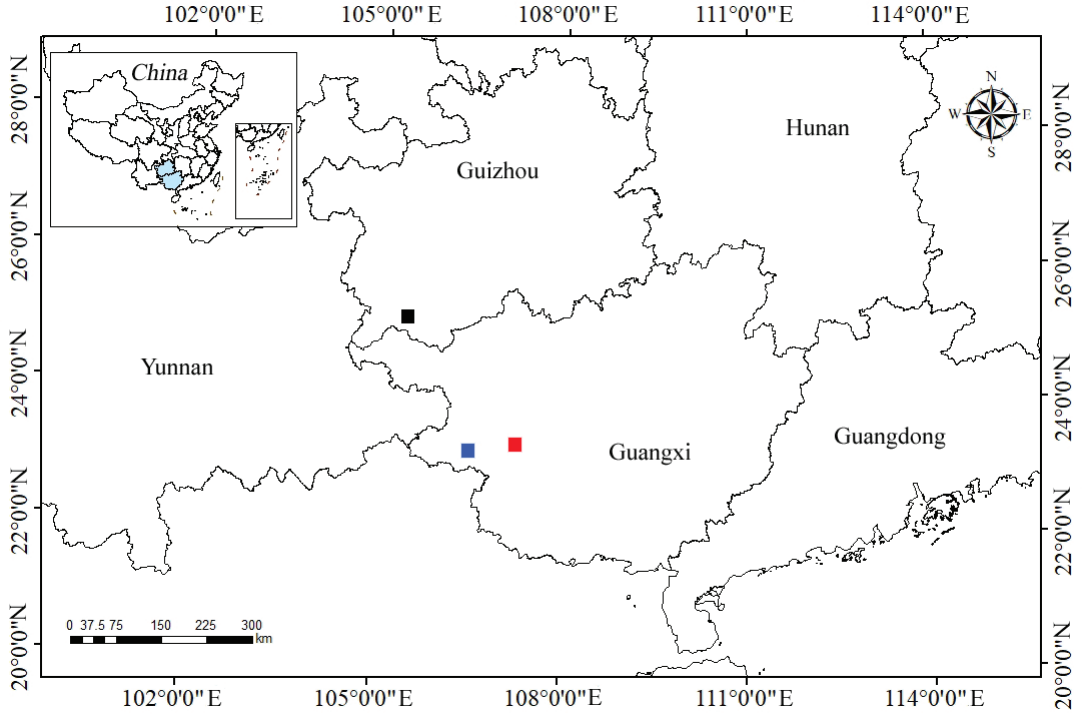
The distribution of similar species of *Balitora*, *B. tiandengensis* sp. nov. (red square), *B. ludongensis* (blue square), and *B. anlongensis* (black square).

***Holotype*** • WGY2024121901 (Fig. 2), 47.3 mm total length (TL), 38.8 mm standard length (SL), collected by You Nong & Gui-Yuan Wei on 19 December 2024 in a karst cave nearby Duoru Village, Dukang Town, Tiandeng County, Guangxi Zhuang Autonomous Region, China (23°03'45.32"N, 106°59'44.81"E, 545 m; Fig. 5). ***Paratypes*** • Four specimens, WGY2024121902–WGY2024121905, 43.6–55.3 mm TL, 33.9–47.1 mm SL, collected together with the holotype.

##### Diagnosis.

*Balitora tiandengensis* sp. nov. can be distinguished from other congeners by the following combination of characters: (1) two pairs of maxillary barbels; (2) dorsal fin rays iii, 9; (3) pectoral fin rays v, 12; (4) pelvic fin rays ii, 7; (5) anal fin rays i, 6; (6) lateral-line scales 69–75; and (7) 6–7 indistinctly separated transversely oval blotches on the dorsal side.

##### Description.

Morphological data of the five specimens of the *Balitora tiandengensis* sp. nov. are provided in Table 2. Body elongated and sub-cylindrical, posterior portion gradually compressed from dorsal fin to caudal-fin base, with deepest body depth anterior to dorsal-fin origin, deepest body depth 15.4–16.3% of SL. Dorsal profile slightly convex from snout to dorsal-fin insertion, then straight from posterior portion of dorsal-fin origin to caudal-fin base. Pelvic profile flat. Head blunt and depressed, head length (HL) 18.7–19.4% of SL and greater than head width, head width greater than depth (head width/head depth = 1.4). Snout short, oblique, and blunt, length 40.9–43.5% HL. Interorbital space wide and flat. Mouth inferior, small and curved, mouth corner situated below anterior nostril, upper and lower lips smooth and fleshy. Relatively shallow preoral groove present between rostral cap and upper lip, extending across corners of mouth. Mouth width 40.0–43.5% of head width. Rostral cap around upper lip divided into three lobes, median one largest, slightly curved. Four pairs of barbels: two pairs of rostral barbels, short, outer rostral barbel longer than inner one; two pairs of maxillary barbels, short, situated at corner of mouth: outer maxillary barbels longer than inner ones. Mouth inferior, small and curved, mouth corner situated below anterior nostril, lips without complex folding or thickening, maintaining a relatively thin and simple structure, upper and lower lips smooth and fleshy. Upper and lower lips connected at corner of mouth, and upper lip with a row of inconspicuous papillae, and lower lip thin. Lower jaw with radiate ridges on its surface. Two longitudinal fleshy ridges on mid-chin. Anterior and posterior nostrils closely set, anterior nostril without any elongated barbel-like tip. Eyes small, 12.7–17.1% HL. Gill opening small, gill rakers not developed.

**Table 2.** Morphological characters and measurement data (mm) of the new species *Balitora tiandengensis* sp. nov. and *B. anlongensis* (data from Luo et al. 2023) and *B. ludongensis* (data from Liu et al. 2012b).

Characters	*Balitora tiandengensis* sp. nov.						*B. anlongensis* (holotype GZNU20230215007)	*B. ludongensis* (holotype KIZ 2008008640)
	WGY2024121901 (holotype)	WGY2024121902	WGY2024121903	WGY2024121904	WGY2024121905	Mean ± SD		
Dorsal-fin rays	iii,9	iii,9	iii,9	iii,9	iii,9	-	iii, 8½	iii,8
Pectoral-fin rays	v,12	v,12	v,12	v,12	v,12	-	viii, 11	vii,12
Pelvic-fin rays	ii,7	ii,7	ii,7	ii,7	ii,7	-	ii, 9	ii,7
Anal-fin rays	i,6	i,6	i,6	i,6	i,6	-	iii, 5½	ii,5
Caudal-fin rays	ii,19	ii,19	ii,19	ii,19	ii,19	-	17	-
Lateral-line pores/scales	69	72	70	69	75	71.5 ± 2.6	68	70
Total length	47.3	45.6	55.3	48.9	43.6	48.4 ± 5.1	60.4	-
Standard length	38.8	36.1	47.1	39.6	33.9	39.2 ± 5.8	47.9	63.2
Body depth	6.0	5.9	5.9	5.8	5.7	5.8 ± 0.1	5.2	12
Body width	6.5	6.3	6.1	6.2	6.0	6.2 ± 0.1	6.5	12.4
Head length	7.0	7.1	6.8	6.9	6.8	6.9 ± 0.1	10.3	12.6
Head depth	4.5	4.6	4.5	4.3	4.4	4.5 ± 0.1	4.9	8.4
Head width	6.9	7	7.1	6.8	7.0	7 ± 0.1	8.7	10.7
Pre-anterior distance	3.1	3.0	3.0	2.9	3.0	3 ± 0.1	3.5	-
Distance between anterior nostrils	2.5	2.3	2.4	2.5	2.4	2.4 ± 0.1	2.8	-
Distance between posterior nostrils	4.0	4.0	4.0	3.8	3.9	3.9 ± 0.1	3.8	-
Distance between anterior and posterior nostrils	2.2	2.0	2.1	2.1	2.0	2.1 ± 0.1	2	-
Snout length	3.0	2.9	2.8	3.0	2.8	2.9 ± 0.1	5.9	6.9
Upper jaw length	2.0	1.9	2.1	2.0	2.0	2 ± 0.1	2.8	-
Lower jaw length	1.1	1.0	0.9	1.0	1.0	1 ± 0.1	2	-
Mouth width	3.1	2.8	2.7	2.9	2.8	2.8 ± 0.1	3.6	-
Eye diameter	1.1	0.9	1.1	0.9	1.2	1 ± 0.2	1.3	1.9
Interorbital distance	4.1	3.8	3.9	4.2	4.1	4 ± 0.2	4.7	5.1
Predorsal length	16.4	17.3	16.2	17.1	15.8	16.6 ± 0.7	22.3	28.8
Dorsal-fin base length	6.2	5.8	5.6	6.1	5.9	5.9 ± 0.2	7.4	-
Dorsal-fin length	7.1	7.2	6.9	7.1	6.4	6.9 ± 0.4	10.9	-
Pectoral-fin length	8.1	7.8	8.3	7.5	7.2	7.7 ± 0.5	11.7	15.4
Pectoral-fin base length	4.4	3.5	3.2	4.1	3.2	3.5 ± 0.4	4.9	-
Pre-pectoral length	6.1	5.8	5.5	6.1	5.2	5.7 ± 0.4	9.3	-
Pelvic-fin length	6.2	5.8	5.9	5.8	5.8	5.8 ± 0.1	10	-
Pelvic-fin base length	2.8	2.5	2.6	2.7	2.6	2.6 ± 0.1	3.4	-
Pre-pelvic length	16.4	15.3	16.1	15.6	15.1	15.5 ± 0.4	22.6	-
Anal-fin length	6.4	5.8	5.9	6.2	5.9	6 ± 0.2	8.7	-
Anal-fin base length	3.6	3.4	3.5	3.5	3.5	3.5 ± 0.1	3.9	-
Pre-anal length	30.2	28.1	29.4	28.3	29.8	28.9 ± 0.8	34.8	47.3
Distance between origin of pectoral fin and origin of ventral fin	12.1	11.8	11.3	12.5	11.7	11.8 ± 0.5	10	-
Distance between origin of ventral fin and origin of anal fin	12.2	12.3	11.6	12.4	11.4	11.9 ± 0.5	10.6	-
Distance between end of Anal fin and anus	9.2	8.4	8.3	9.1	8.2	8.5 ± 0.4	1.8	-
Caudal peduncle length	8.5	8.1	8.2	8.2	8.1	8.2 ± 0.1	8.6	-
Caudal peduncle depth	8.5	9.2	8.3	9.1	8.6	8.8 ± 0.4	3.1	-
Inner maxillary barbel length	2.3	2.5	2.3	2.2	2.3	2.3 ± 0.1	1.4	-
Outer maxillary barbel length	2.1	2.2	2.1	2.1	2.1	2.1 ± 0.1	1.1	3.6
Inner rostral barbel length	1.2	1.1	1.3	1.2	1.1	1.2 ± 0.1	1.4	-
Outer rostral barbel length	2.3	2.2	2.1	2.2	2.1	2.2 ± 0.1	1.9	-

Dorsal fin iii, 9; anal fin i, 6; pectoral fin v, 12; pelvic fin ii, 7; and 19 branched caudal-fin rays. Dorsal fin long, 16.7–19.4% of SL, nearly equal to head length, distal margin truncated, origin anterior to pelvic fin insertion, situated slightly anterior to midpoint between snout tip and the caudal-fin base, first branched ray longest, shorter than HL, tip of the dorsal fin extending to the vertical of the anus. Pectoral fin elongated and developed, distal margin rounded, pectoral fin length approximately equal than HL, 18.4–20.5% of SL, tip of the pectoral fin extends backward beyond 54.0%–60.0% of the distance between the origin of the pectoral fin and the origin of the pelvic fin, without reaching to the pelvic fin-origin. Pelvic fin moderately developed, distal margin rounded, pelvic fin length slightly shorter than HL, 15.1–16.7% of SL, vertically aligned with the fourth branched ray of the dorsal fin, pelvic fin origin closer to the snout tip than the caudal-fin base and closer to the anal fin origin than the snout tip, tips of the pelvic fin reaching to the anus. Anus 72.5%-73.3% distance from posterior end of the pelvic fin base to the anal fin origin. Anal fin short, 15.0–16.7% of SL, distal margin truncated, origin close to the anus and far from the caudal-fin base, spacing ~ 2.5 mm, tips of the anal fin extending backwards and not reaching caudal-fin base, distance between the end of the anal fin and the anus 7.5–9 × the eye diameter. Caudal fin deeply forked, upper lobe equal in length to the lower one, tips pointed, caudal peduncle length 8.1 mm, caudal peduncle depth 2.8 mm, without adipose crests along both dorsal and ventral sides.

Body smooth, covered with thin scales all over except for on the ventral side, head, and fins. Lateral line complete and straight, with 69–75 lateral line scales, exceeding the tip of the pectoral fin and reaching the base of the caudal fin.

##### Sexual dimorphism.

No sexual dimorphism was observed based on the present specimens of *Balitora tiandengensis* sp. nov.

##### Coloration in life.

Dorsal and lateral side of body brown, while ventral side white with slightly pinkish. Dorsal side of head blackish-brown, usually with black irregular blotches. Dorsal side of body with six or seven transverse black round blotches encircled by yellow interspace, usually two or three blotches in front of dorsal-fin origin, one or two on dorsal-fin base and three or four behind. Dorsal fin almost hyaline with discontinuous black spots. Pectoral and pelvic fin bases pale gray from dorsal view; dorsal side of pectoral and pelvic fin rays blackish spots, membrane lighter; distal tip of pectoral and pelvic fin hyaline and colorless. Anal fin hyaline with a pale blackish bar in the middle. Caudal fin almost hyaline with blackish spots. Lateral line of the body from the posterior to the eye to the base of the caudal fin pale grayish-yellow. After being moved from inside the cave to outside, the body pigmentation lightened in ~ 6 hours while it was alive.

##### Coloration in alcohol.

After being fixed in 10% formalin and stored in 70% ethanol, the body pigmentation deepened. Other parts of the body similar to the living condition.

##### Distribution.

*Balitora tiandengensis* sp. nov. is only known from the type locality, a karst cave near Duoru Village, Dukang Town, Tiandeng County, Guangxi, China at an elevation of 545 m (Fig. 5).

##### Ecology.

Within this cave, *Balitora tiandengensis* sp. nov. co-occurred with catfish (*Silurus* sp). Around the cave, the arable land was farmed to produce tea (*Ilex latifolia* Thunb.).

##### Etymology.

The specific epithet tiandengensis is in reference to the type locality of the new species: Duoru Village, Dukang Town, Tiandeng County, Guangxi Zhuang Autonomous Region, China. We propose the common English name “Tiandeng cave loach” and the Chinese name “tiān děng pá qiū (天等爬鳅)”.

## Discussion

The new species is assigned to the genus *Balitora* based on the combination of the following diagnostic characters (Kottelat 1988; Kottelat and Chu 1988): (1) body strongly depressed; head and abdomen ventrally flattened; (2) mouth inferior, arched, with both jaws covered by a horny sheath; (3) rostral flap divided into three lobes, the lips exhibit no complex folding or thickening, maintaining a relatively thin and simple structure; upper lip with a row of inconspicuous papillae; (4) two pairs of maxillary barbels; (5) gill-openings extending on the ventral surface of head; (6) two unbranched pelvic rays, eight branched pelvic rays; (7) five unbranched pectoral rays, ten branched pectoral rays; and (8) adhesive pads present on ventral surface of the 8–11 anterior most pectoral rays and three or four anteriormost pelvic rays.

Our molecular phylogenetic analysis, based on COI+Cyt b+RAG1+IRBP+EGR2B genes, confirms *Balitora tiandengensis* as a distinct lineage within *Balitora*, supported by high bootstrap values (100%) and genetic divergence metrics (uncorrected p-distance ≥ 0.6% from congeners). This aligns with prior studies demonstrating that *Balitora* is non-monophyletic (Luo et al. 2023; Lei et al. 2025a), with species clustering into three clades: one containing the type species *B. brucei*, another comprising *B. kwangsiensis* (formerly Sinohomaloptera), and a third including *B. tiandengensis*, *B. anlongensis*, and *B. ludongensis*.

Morphologically, *Balitora tiandengensis* is distinguished from congeners by its unique combination of traits: (1) two pairs of maxillary barbels; (2) dorsal fin rays iii, 9; (3) pectoral fin rays v, 12; (4) pelvic fin rays ii, 7; (5) anal fin rays i, 6; (6) lateral-line scales 69–75; and (7) six or seven indistinctly separated transversely oval blotches on the dorsal side. These features contrast with *B. ludongensis*, with which it shares a similar distribution but differs in having more dorsal fin rays, anal fin rays, pelvic fin rays, and lateral line scales (Liu et al. 2012b). Such morphological plasticity within the genus underscores the need for integrative taxonomic approaches, as reliance on single traits (e.g., barbel number) has historically led to misclassifications (e.g., *B. pengi* and *B. tchangi* were initially placed in Hemimyzon due to overlapping morphological characters; Kottelat 1988).

*Balitora tiandengensis* sp. nov. differs from *B. annamitica*Kottelat 1988, *B. brucei*, *B. burmanica*, *B. chipkali*, *B. eddsi* Conway & Mayden, 2010, *B. jalpalli*, *B. lancangjiangensis*, *B. laticauda*, *B. meridionalis*, *B. mysorensis*, *B. nantingensis*, *B. elongata*, *B. tchangi*, and *B. yingjiangensis* based on the presence of two maxillary barbels at each corner of the mouth (vs 1). *Balitora tiandengensis* sp. nov. differs from *B. kwangsiensis*, *B. longibarbata*, and *B. ludongensis* based on the dorsal fin rays (iii, 9 vs iii, 8). *Balitora tiandengensis* sp. nov. can be further distinguished from *B. brucei*, *B. burmanica*, *B. chipkali*, *B. jalpalli*, *B. kwangsiensis*, *B. lancangjiangensis*, *B. laticauda*, *B. longibarbata*, *B. ludongensis*, *B. meridionalis*, *B. nantingensis*, and *B. elongata* based on dorsal fin rays (iii, 9 vs iii, 8). *Balitora tiandengensis* sp. nov. can be further distinguished from *B. brucei*, *B. burmanica*, *B. chipkali*, *B. jalpalli*, *B. kwangsiensis*, *B. lancangjiangensis*, *B. laticauda*, *B. longibarbata*, *B. ludongensis*, *B. meridionalis*, *B. nantingensis*, and *B. elongata* based on dorsal fin rays (iii, 9 vs iii, 8) (Table 3).

**Table 3.** Comparison of the diagnostic characters of the new species described here with those selected for the 19 species of the genus *Balitora*. The morphological data of these species comes from relevant literature (Liu et al. 2012b; Luo et al. 2023; Lei et al. 2025b).

ID	Species	Maxillary barbels	Dorsal fin rays	Anal fin rays	Pectoral fin rays	Pelvic fin rays	Lateral line scales	Dorsal black spot
1	*Balitora tiandengensis* sp. nov. (*n* = 5)	2	iii, 9	i, 6	v, 12	ii, 7	69–75	6–7
2	*B. anlongensis* (*n* = 11)	2	iii, 8½	iii, 5½	viii, 11	iii, 9	66–68	6–7
3	*B. annamitica*	1	?, 12	?, 8	viii–x, 8	-	61–62	-
4	*B. brucei*	1	iii, 8	i, 5	ix, 11	ii, 9	65–69	-
5	*B. burmanica* (*n* = 4)	1	iii, 8	i, 5	ix, 11	ii, 8	70–72	-
6	*B. chipkali*	1	iii, 8	iii, 5	iii–ix, 11–12	ii, 9	66–68	7
7	*B. eddsi*	1	iii, 9	iii, 5–7	vi, 10–12	ii, 8–9	66–67	Without
8	*B. elongata*	1	iii, 8	ii, 5	x, 10–12	iii, 8	67	7
9	*B. jalpalli*	1	iii, 8	ii, 5	ix, 10–11	ii, 8–9	64–66	9
10	*B. kwangsiensis* (*n* = 6)	2	iii, 8	ii, 5	vi–viii, 10–13	ii, 8	61–65	6–8
11	*B. lancangjiangensis*	1	iii, 8	ii, 5	viii, 10–12	ii, 8–9	68–70	7–8
12	*B. laticauda*	1	iii, 8	iii, 5	viii–ix, 10–11	ii, 8–9	66–68	10
13	*B. longibarbata* (*n = 8*)	2	iii, 8	ii, 5	viii–x, 11–14	ii, 9–11	74–76	8–9
14	*B. ludongensis* (*n = 7*)	2	iii, 8	ii, 5	vi–vii, 11–12	ii, 6–7	69–74	6–9
15	*B. meridionalis*	1	?, 11	?, 8	?, 9–10	-	-	-
16	*B. mysorensis*	1	iii, 8–9	ii, 5	viii-ix 10–12	ii, 8–9	68–69	-
17	*B. nantingensis* (*n = 4*)	1	iii, 8	ii, 5	viii–x, 9–12	ii, 9	59–64	-
18	*B. elongata*	1	iii, 8	ii, 5	x, 10–12	iii, 8	67	7
19	*B. tchangi*	1	iii, 7	ii, 5	xii, 13	v, 13	74	8
20	*B. yingjiangensis* (*n = 11*)	1	iii, 7	iii, 5½	ix, 14	ii, 9	63–67	-

**Table 4.** Uncorrected p-distance (%) amongst 11 *Balitora* spp., based on COI, Cyt b, RAG1, IRBP, EGR2B genes.

	*Balitora anlongensis*	*Balitora annamitica*	*Balitora brucei*	*Balitora chipkali*	*Balitora elongata*	*Balitora jalpalli*	*Balitora kwangsiensis*	*Balitora laticauda*	*Balitora ludongensis*	*Balitora mysorensis*
*Balitora anlongensis*										
*Balitora annamitica*	0.95									
*Balitora brucei*	0.58	0.33								
*Balitora chipkali*	1.89	0.77	0.52							
*Balitora elongata*	1.26	0.58	0.01	1.1						
*Balitora jalpalli*	0.58	0.3	0.55	0.06	0					
*Balitora kwangsiensis*	1.39	0.7	-	1.35	1.2	-				
*Balitora laticauda*	1.88	0.8	0.49	0.56	1.11	0.11	1.28			
*Balitora ludongensis*	1.98	0.84	0.64	1.86	1.11	0.67	1.1	1.88		
*Balitora mysorensis*	1.78	0.83	0.4	1.67	1.12	0.49	1.26	1.54	1.72	
*Balitora tiandengensis*	2.51	0.89	0.63	2.23	1.57	0.6	1.62	2.25	1.18	2.24

*Balitora tiandengensis* sp. nov. resembles *B. ludongensis* but can be further distinguished based on the dorsal fin rays (iii, 9 vs iii, 8), anal fin rays (i, 6 vs ii, 5), pectoral fin rays (v, 12 vs vi–vii, 11–12), lateral line scales (69–75 vs 66–68). Its percentage of body depth/SL (15.5 vs 18.9), body width/SL (16.8 vs 19.6), head length/SL (18.0 vs 20.0), predorsal length/SL (42.3 vs 45.6), preanal length/SL (77.8 vs 74.9), head depth/head length (64.3 vs 66.3), head width/head length (98.6.0 vs 85.1), eye diameter/head length (15.7 vs 14.8) (Table 3).

*Balitora tiandengensis* sp. nov. is similar to *B. anlongensis* but it can be distinguished from the latter by the combination of the following morphological characters: dorsal fin rays (iii, 9 vs iii, 8), anal fin rays (i, 6 vs iii, 5), pectoral fin rays (v, 12 vs viii, 11), pelvic fin rays (ii, 7 vs iii, 9), lateral line scales (69–75 vs 66–68). Its standard length (33.9–47.1 mm vs 39.8–48.2 mm), head length (6.8–7.1 mm vs 8.4–10.6 mm), snout length (2.8–3.0 mm vs 4.9–6.1 mm), outer maxillary barbel length (2.1–2.2 mm vs 0.9–1.5 mm) (Table 3).

The discovery of *Balitora tiandengensis* sp. nov. significantly advances our understanding of the systematics and biogeography of the genus *Balitora*, a group of rheophilic fishes adapted to high-gradient stream environments across south and southeast Asia (Kottelat and Chu 1988; Kottelat 1988; Nguyen 2005; Conway and Mayden 2010; Liu et al. 2012b; Luo et al. 2023; Lei et al. 2025a, 2025b).

The distribution of *B. tiandengensis* extends the known range of *Balitora* eastwards. This finding aligns with recent discoveries of cave-adapted *Balitora* in Guizhou (Luo et al. 2023), suggesting that the genus has undergone multiple dispersal events across mountain ranges.

*Balitora tiandengensis* inhabits in cave with fast-flowing, oxygen-rich waters, where it likely plays a role in controlling algal biofilms and invertebrate populations. However, similar to other *Balitora* species, *B. tiandengensis* may exhibit low dispersal capacity due to its specialized morphology, increasing its susceptibility to local extinctions. Future research should assess population genetics and habitat requirements to inform management strategies.

## Supplementary Material

XML Treatment for Balitora tiandengensis

## Additional information

### Use of AI

No use of AI was reported.

### Author ORCIDs

You Nong https://orcid.org/0000-0001-7004-0946

Yuan Fang https://orcid.org/0009-0001-3637-0762

Jin-Yue Li https://orcid.org/0000-0003-4920-4322

Qiu-Jun Wei https://orcid.org/0009-0002-5799-6323

Chuan-Gui Xu https://orcid.org/0009-0000-6263-3821

Gui-Yuan Wei https://orcid.org/0000-0003-0652-1213

### Data availability

All of the data that support the findings of this study are available in the main text or Supplementary Information.

## Supplementary materials

Supplementary material 1 Primer sequences Data type xlsx https://binary.pensoft.net/file/1523038This dataset is made available under the Open Database License (http://opendatacommons.org/licenses/odbl/1.0/). The Open Database License (ODbL) is a license agreement intended to allow users to freely share, modify, and use this Dataset while maintaining this same freedom for others, provided that the original source and author(s) are credited. You Nong, Yuan Fang, Jin-Yue Li, Qiu-Jun Wei, Chuan-Gui Xu, Gui-Yuan Wei
